# Alterations in Circulating Fatty Acid Are Associated With Gut Microbiota Dysbiosis and Inflammation in Multiple Sclerosis

**DOI:** 10.3389/fimmu.2020.01390

**Published:** 2020-07-07

**Authors:** Marina Saresella, Ivana Marventano, Monica Barone, Francesca La Rosa, Federica Piancone, Laura Mendozzi, Alessia d'Arma, Valentina Rossi, Luigi Pugnetti, Gabriella Roda, Eleonora Casagni, Michele Dei Cas, Rita Paroni, Patrizia Brigidi, Silvia Turroni, Mario Clerici

**Affiliations:** ^1^IRCCS Fondazione Don Carlo Gnocchi, Milan, Italy; ^2^Microbial Ecology of Health Unit, Department of Pharmacy and Biotechnology, University of Bologna, Bologna, Italy; ^3^Departments of Pharmaceutical Sciences, University of Milan, Milan, Italy; ^4^Health Sciences, University of Milan, Milan, Italy; ^5^Pathophysiology and Trasplantation, University of Milan, Milan, Italy

**Keywords:** short-chain fatty acids (SCFAs), butyric acid, caproic acid, gut microbiota, dysbiosis, cytokines, T lymphocytes, multiple sclerosis

## Abstract

**Background:** Butyric acid (BA) is a short-chain fatty acid (SCFA) with anti-inflammatory properties, which promotes intestinal barrier function. Medium-chain fatty acids (MCFA), including caproic acid (CA), promote TH1 and TH17 differentiation, thus supporting inflammation.

**Aim:** Since most SCFAs are absorbed in the cecum and colon, the measurement of BA in peripheral blood could provide information on the health status of the intestinal ecosystem. Additionally, given the different immunomodulatory properties of BA and CA the evaluation of their serum concentration, as well as their ratio could be as a simple and rapid biomarker of disease activity and/or treatment efficacy in MS.

**Methods:** We evaluated serum BA and CA concentrations, immune parameters, intestinal barrier integrity and the gut microbiota composition in patients with multiple sclerosis (MS) comparing result to those obtained in healthy controls.

**Results:** In MS, the concentration of BA was reduced and that of CA was increased. Concurrently, the microbiota was depleted of BA producers while it was enriched in mucin-degrading, pro-inflammatory components. The reduced serum concentration of BA seen in MS patients correlated with alterations of the barrier permeability, as evidenced by the higher plasma concentrations of lipopolysaccharide and intestinal fatty acid-binding protein, and inflammation. Specifically, CA was positively associated with CD4+/IFNγ+ T lymphocytes, and the BA/CA ratio correlated positively with CD4+/CD25^high^/Foxp3+ and negatively with CD4+/IFNγ+ T lymphocytes.

**Conclusion:** The gut microbiota dysbiosis found in MS is possibly associated with alterations of the SCFA/MCFA ratio and of the intestinal barrier; this could explain the chronic inflammation that characterizes this disease. SCFA and MCFA quantification could be a simple biomarker to evaluate the efficacy of therapeutic and rehabilitation procedures in MS.

## Introduction

Multiple sclerosis (MS) is a chronic disease of the central nervous system (CNS) characterized by demyelination and mediated by auto-reactive immune processes directed against neural tissues. Inflammation drives and accompanies MS, and this disease has repeatedly been shown to be characterized by augmented activation of TH1 and TH17 lymphocytes and downregulation of Treg cells. The etiopathogenesis of MS is still only partly understood, but a number of recent publications suggest that alterations of the intestinal microbiota play a role, even if the underlying mechanisms are unclear (1–4). Compared to healthy individuals, the gut microbial ecosystem of MS patients is indeed enriched with *Methanobrevibacter* (5), an archaeal genus associated with inflammatory and autoimmune diseases (6), and *Akkermansia*, a mucin-degrading bacterium capable of favoring pro-inflammatory T lymphocyte responses,7 but is depleted of *Parabacteroides distasonis* (7), bacterial species that support T cell differentiation into Treg cells (8). An additional body of data indicates that the relative abundance of the *Lachnospiraceae* and *Ruminococcaceae* families, which include short-chain fatty acid (SCFA) producers, as well as *Bacteroides fragilis, Butyricimonas*, and *Prevotella* is considerably reduced in MS (4, 5, 9–11). The hypothesis that these alterations play a role in the pathogenesis of MS has recently been reinforced by results showing that diet-induced changes in the gut microbiota resulted in a significant increase in *Lachnospiraceae* that correlated with an augmented expression of anti-inflammatory immune cells and a significant reduction of the relapse rate, as well as of the expanded disability status score (EDSS) (12).

Butyric, acetic and propionic acids are the main SCFAs, i.e., carboxylic acids containing 2–5 carbon atoms, which are produced in the proximal colon by bacterial fermentation of non-digestible carbohydrates (13, 14). Butyric acid (BA), in particular, is endowed with immunomodulatory properties that are mediated by histone deacetylase (HDAC) inhibition (15–17) or through the activation of the metabolite-sensing G protein-coupled receptors GPR41, GPR43, and GPR109A (18–23). Thus, SCFA-mediated stimulation of Treg cell differentiation is driven by HDAC inhibition, while IL-10 production is mediated by GPR41 or GPR43 activation (23). Notably, recent results have shown that SCFAs could mediate the activation of pro-inflammatory immune circuits as well, depending on the cytokine milieu and immunological context (23–26). In contrast to SCFAs, medium-chain fatty acids (MCFAs) mostly derive from the diet, even if the liver contributes to their production via peroxisomal beta-oxidation of long-chain fatty acids. MCFAs control carbohydrate and lipid tissue metabolism as well as the production of mitochondrial energy (27) and antagonize the anti-inflammatory activities of SCFAs, as they favor TH1 and TH17 differentiation. The immunological roles of BA and MCFAs, particularly of caproic acid (CA), have been confirmed in animal models (28, 29). In these models, BA has been shown to stimulate the maturation of fully functional Treg cells, and MCFAs have been confirmed to have an effect that antagonizes that of SCFAs, as they enhance TH1 and TH17 cell differentiation (29). In addition to its direct anti-inflammatory effect, BA also downregulates inflammation secondarily to its pivotal role in maintaining the integrity of the gastrointestinal (GI) epithelial barrier through regulation of mucus production and tight junction expression (13, 30, 31). Therefore, BA prevents alterations of intestinal permeability and the consequent translocation of lipopolysaccharide (LPS) from the gut lumen to the peripheral blood. As repeatedly demonstrated in the setting of other diseases, such as HIV infection (32), increased amounts of LPS in peripheral blood drive chronic inflammation by binding to toll-like receptor (TLR) 4, an activatory receptor expressed by lymphoid cells.

Since over 90% of SCFAs are absorbed in the cecum and colon, and only 5–10% of them are excreted in the feces (33–36), the measurement of BA in peripheral blood could provide information on the health status of the intestinal ecosystem (37). Additionally, given the many beneficial functions of BA as an immune-modulator, its monitoring could work as a simple and rapid biomarker of disease activity and/or treatment efficacy in MS. Herein, we analyzed serum concentrations of BA and CA, as well as immune parameters and parameters of integrity of the GI epithelial barrier, and profiled the gut microbiota in MS patients and healthy controls (HC).

## Methods

### Individuals Enrolled in the Study

Thirty-eight patients with a diagnosis of relapsing-remitting (RR) or secondary-progressive (SP) Multiple Sclerosis (MS) (20 females and 18 males; median age = 47 years, IQ = 42–57) who are followed by the Multiple Sclerosis Rehabilitation Unit of the Don Carlo Gnocchi Foundation in Milan, Italy, were enrolled in the study. Inclusion criteria were age >18 years and disease stability for >6 months prior to enrollment. Main exclusion criteria were: (1) changes in disease-modifying treatment (DMD) in the 6 months prior to enrollment; (2) use of corticosteroids in the 6 months prior to enrollment; (3) presence of significant co-morbidities, including arterial hypertension, cerebrovascular disorders, heart or pulmonary diseases, diabetes, endocrine, gastrointestinal, or psychiatric diseases. Patients were following a free diet (western type) as determined by an expert dietician through interviews. At the time of enrolment and of biological sampling none of the patients was using antibiotics; none of the patients had used any antibiotic in the 3 months preceding enrolment and biological sampling. Stable disease was diagnosed on the basis of brain and spinal cord magnetic resonance imaging (MRI) with gadolinium showing no areas of enhancement at the time of enrolment. Median disease duration was 19 years (range: 15–24 years); disability level, as assessed by the median Kurtze Expanded Disability Status Scale (EDSS) score was 5.3 (range: 3–6). Finally, a group of 38 sex- and age-matched HC (median age = 48 years; range 33–62; 20 females and 18 males) was enrolled as well in the study. HC were also following a free, western style diet and had not taken antibiotics or probiotics in the 3 months prior to sampling. Italian controls have been specifically selected to reduce any bias related to lifestyle or, more generally, to the geographical effect, for which the strongest associations with microbiota variation have recently been shown (38). The Ethics Committee of the Don Carlo Gnocchi Foundation approved the study protocol; all the enrolled subjects signed an informed consent. The clinical and demographic characterization of the MS patients enrolled in the study is presented in Supplementary Table 1.

### Serum

Serum was collected in vacutainer tubes containing serum separator (Becton Dickinson & Co., Rutherford, NJ, USA), centrifuged at 3,000 rpm for 10 min, and stored at −80°C until use.

### Analysis by LC-MS/MS of Serum Fatty Acids

Butyric and caproic acids were extracted and analyzed by LC-MS/MS according to the protocol described in Dei Cas (39). Briefly, serum (50 μl) was deproteinized by isopropanol (100 μl) and fatty acids derivatized with nitrophenylhydrazine. The extract was analyzed on an HPLC Dionex 3000 UltiMate system (Thermo Fisher Scientific, MA, USA) coupled to a tandem mass spectrometer AB Sciex 3200 QTRAP (AB Sciex, Milan, Italy) operated by multiple reaction monitoring under negative electrospray ionization.

### Blood Sample Collection and Cell Separation

Whole blood (10 ml) was collected in vacutainer tubes containing ethylenediaminetetraacetic acid (EDTA) (Becton Dickinson & Co.). Peripheral blood mononuclear cells (PBMCs) were separated on lympholyte separation medium (Cedarlane, Hornby, Ontario, CA, USA) and washed twice in PBS at 1,500 rpm for 10 min; viable leukocytes were determined with Bio-Rad TC20 Automated Cell Counter (Bio-Rad Laboratories, Hercules, CA, USA).

### Intracellular Cytokine and Transcription Factor Staining in PBMCS

Lymphocyte subsets were analyzed in freshly isolated PBMCs that were incubated for 30 min at 4°C in the dark with Phycoerythrin-Cyanine-7 (PC7)-labeled anti-CD4 (clone SFCI12T4D11, mouseIgG_1_, Beckman-Coulter, Brea, CA, USA) or Phycoerythrin-Texas Red (ECD)-labeled anti-CD25 (clone B1.49.9, mouse IgG_2a_, Beckman-Coulter). After incubation cells were washed, permeabilized with a Cell Permeabilization kit (FIX & PERM kit, eBioscience) and incubated for 30 min at 4°C in the dark with either anti-IL-10 (clone JES9D7, mouse IgG1, R&D Systems), or anti-IFNγ (clone 25723, mouse IgG_2b_, R&D Systems) PE-labeled antibodies. The PC-5-labeled-anti-IL-17 (clone BL168, mouse IgG_1k_, Biolegend) and the Alexa Fluor 488-labeled-anti-Foxp3 (clone 1054C, rabbit IgG, R&D) antibodies were used as well.

### Flow Cytometry Analysis

PBMCs were analyzed to identify regulatory T cells (Tregs: CD4+CD25++FOXP3+), TH1 (CD4+IFNγ+), TH17 (CD4+IL-17+) and inducible regulatory T cells (TH_R_1: CD4+IL-10+) using a Beckman-Coulter GALLIOS flow cytometer equipped with a 22 mW Blue Solid State Diode laser operating at 488 nm and with a 25 mW Red Solid State Diode laser operating at 638 nm, and interfaced with Kaluza analysis software. Two hundred thousand cells were acquired and gated on lymphocyte FSC and SSC properties. Isotype control or single fluorochrome-stained preparations were used for color compensation.

### Microbial Translocation and Gastrointestinal Barrier Function

LPS was measured in plasma with the LAL Chromogenic Endpoint Assay (Hycult biotechnology, Uden, The Netherlands); I-FABP was measured with an ELISA kit (CUSABIO BIOTECH, Newark, DE, USA) according to the manufacturer's instructions.

### Gut Microbiota Analysis

Total bacterial DNA was extracted from stool samples of 35 MS patients, as previously described (40). The V3-V4 hypervariable region of the 16S rRNA gene was PCR-amplified using the 341F and 785R primers (41) with Illumina overhang adapter sequences as previously reported (40) PCR products of about 460 bp were purified using a magnetic bead-based system (Agencourt AMPure XP; Beckman Coulter) and indexed by limited-cycle PCR using Nextera technology. Indexed libraries, further cleaned up as described above, were pooled at equimolar concentrations, denatured and diluted to 6 pmol/l. Sequencing was performed on an Illumina MiSeq platform using the 2 × 250 bp protocol, according to the manufacturer's instructions (Illumina, San Diego, CA, USA). Sequence reads were deposited in the National Center for Biotechnology Information Sequence Read Archive (NCBI SRA; BioProject ID PRJNS633233).

### Bioinformatics and Statistical Analysis

Quantitative data were not normally distributed (Shapiro-Wilk test) and are therefore summarized as median and interquartile range (IQR; 25th and 75th percentiles). Comparisons between groups were performed using a two-tailed Mann-Whitney test for independent samples. Kruskal-Wallis analysis of variance was utilized for each variable. Statistical correlations between the immunological parameters and BA, CA, or the BA/CA ratio were investigated by the Spearman correlation coefficient and 95% confidence limits performed by Fisher's *Z* transformation. Data analysis was performed using the MEDCALC statistical package (MedCalc Software bvba, Mariakerke Belgium).

As for microbiota analysis, paired-end reads were processed using a pipeline combining PANDAseq (42) and QIIME 2 (43). High-quality reads were filtered and clustered into Amplicon Sequence Variants (ASVs) at 99% similarity through an open-reference strategy performed with DADA2 (44). Singleton ASVs were discarded and chimeras were identified using ChimeraSlayer (45) and then filtered out. Taxonomy was assigned using the vsearch classifier (46) against the Greengenes database as a reference (release May 2013). 16S rRNA gene sequencing data of MS patients were compared to publicly available data of age- and sex-matched healthy Italian subjects [20 subjects: MG-RAST ID 17761 (47), 15 subjects: MG-RAST ID 7058 (48)]. Genus-level community composition was generated for all combined cohorts. Alpha diversity was measured using the Shannon and Simpson indices (estimating evenness and richness), while beta diversity was computed based on Jaccard similarity and visualized on a Principal Coordinates Analysis (PCoA) plot. The significance of the separation between the study groups was tested by a permutation test with pseudo-*F* ratios using the function adonis in the R package, vegan (49). Bar plots were built using the packages made4 (50) and vegan. Kendall rank correlation test was used to assess the associations between genus-level relative abundances and EDSS, levels of T lymphocytes, LPS, I-FABP and fatty acids in MS patients. Statistics was performed using R Studio 1.0.44 on R software version 3.3.2 (https://www.r-project.org/) implemented with the packages stats and vegan. *P*-values were corrected for multiple comparisons using the Benjamini–Hochberg method when appropriate. A *p*-value ≤ 0.05 was considered statistically significant.

## Results

### Serum Concentration of Butyric and Caproic Acids

**Figure 1:**
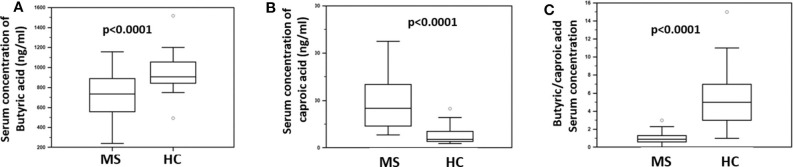
Serum concentration of butyric and caproic acid in MS patients compared to healthy controls. LC-MS/MS analysis of **(A)** serum concentration (ng/ml) of butyric acid and **(B)** caproic acid in Multiple Sclerosis patients (MS) (*n* = 38) and healthy controls (HC) (*n* = 38). **(C)** The butyric/caproic acid ratio. In all panels the boxes stretch from the 25th to the 75th percentile; the line across the boxes indicates the median value; the lines stretching from the boxes indicate extreme values. Outliers are displayed as separate points. Statistical significance is shown.

The concentration of butyric acid (BA) and caproic acid (CA) was analyzed in the serum of all individuals enrolled in the study, by liquid chromatography coupled to mass spectrometry (LC-MS). The results showed that BA was significantly reduced (median: HC = 907 ng/ml; MS = 752 ng/ml; *p* < 0.0001) whereas CA was significantly increased (median: HC = 181 ng/ml; MS = 863 ng/ml; *p* < 0.0001) in MS compared to HC. Accordingly, the BA/CA ratio was significantly reduced in MS (ratio: 0.9) compared to HC (ratio: 5; *p* < 0.0001). These data are shown in Figure 1.

### T Lymphocyte Functional Subpopulations

**Figure 2:**
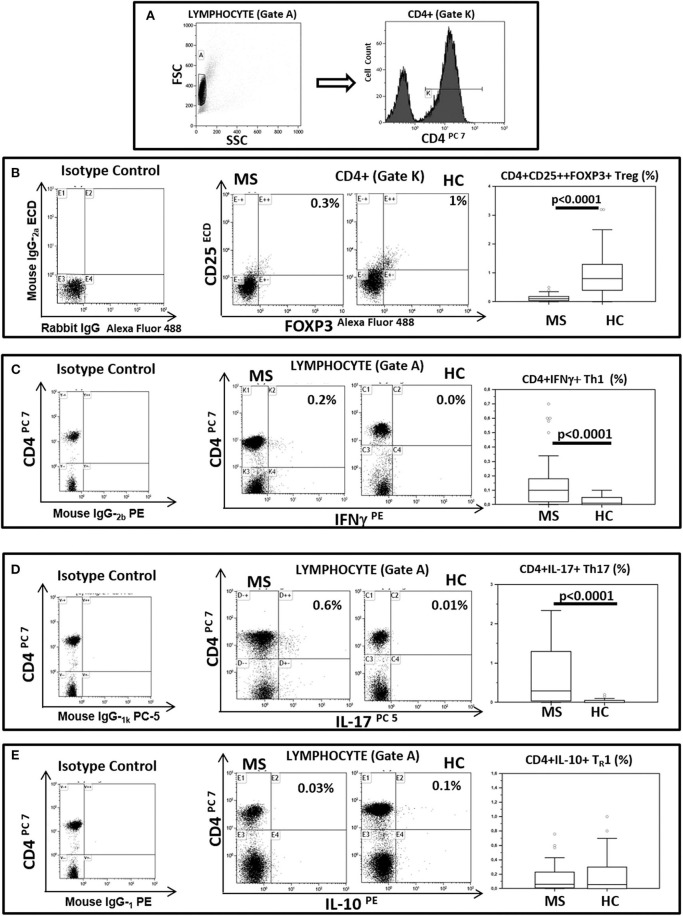
Treg, TH1, TH17, and TH_R_1 CD4+ lymphocyte subsets in peripheral blood of MS patients compared to healthy controls. Representative flow cytometry dot plots results obtained in Multiple Sclerosis patients (MS) (*n* = 38) and healthy controls (HC) (*n* = 38). **(A)** Lymphocytes and CD4+ T lymphocytes (gate strategy), **(B)** Tregs: CD25+ and intracellular FOXP3 expression gated on CD4+T cell (CD4+/CD25^high^/Foxp3+), **(C)** TH1: CD4 and intracellular IFNγ (CD4+/IFNγ+) expression gated on Lymphocyte, **(D)** TH17: CD4 and intracellular IL-17 (CD4+/IL-17+) expression gated on Lymphocyte and **(E)** TH_R_1: CD4 and intracellular IL-10 (CD4+/IL-10+) expression gated on Lymphocyte. In the upper right corner the percentage of Treg, TH1, TH17 and TH_R_1 lymphocytes is presented. Summary results are shown in the bar graphs. The boxes stretch from the 25th to the 75th percentile; the line across the boxes indicates the median value; the lines stretching from the boxes indicate extreme values. Outliers are displayed as separate points. Comparisons between groups were performed using a two-tailed Mann-Whitney test for independent samples. Statistical significance is shown.

BA regulates Treg lymphocyte development whereas CA is known to support TH1 and TH17 differentiation. To verify possible associations between BA and CA and these lymphocyte subsets Treg, TH1 and TH17 were measured in freshly isolated and unstimulated PBMCs from all patients and controls. The results indicated that Treg lymphocytes (CD4+/CD25^high^/Foxp3+) were decreased in MS (median: 0.1%) compared to HC (median: 0. 8%; *p* < 0.0001). On the contrary, both TH1 (CD4+/IFNγ+) (median: HC = 0.01%; MS = 0.1%; *p* < 0.0001) and TH17 (CD4+/IL-17+) (median: HC = 0.0%; MS = 0.3%; *p* < 0.0001) T lymphocytes were significantly increased in MS compared to HC. CD4+/IL-10 positive T lymphocytes were also measured in all individuals; these cells were not significantly different when MS and HC were compared. These results are shown in Figure 2.

### Correlation Between Butyric or Caproic Acid or Their Ratio and T Lymphocyte Subpopulations

**Figure 3:**
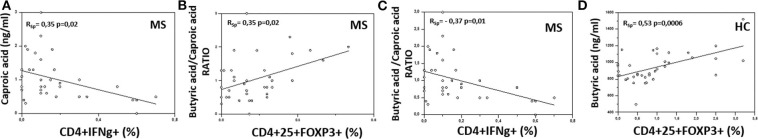
Correlation between the serum concentration of butyric and caproic acid and their ratio and peripheral immune cells in MS and healthy controls. Rank correlation between: **(A)** serum caproic acid concentration and peripheral IFNγ-producing CD4+ T cell percentage, **(B)** butyric acid/caproic acid ratio and Treg (CD4+/CD25^high^/Foxp3+) cell percentage, **(C)** butyric acid/caproic acid ratio and TH1 (CD4+/IFNγ+) cell percentage in Multiple Sclerosis patients (MS) (*n* = 38); **(D)** serum butyric acid concentration and Treg (CD4+/CD25^high^/Foxp3+) cell percentage in healthy controls (HC) (*n* = 38). Statistical correlations were investigated by the Spearman correlation coefficient and 95% confidence limits performed by Fisher's *Z* transformation. Statistical significance and Spearman's coefficient of rank correlation (R_Sp_) are shown.

Possible correlations between the serum concentration of BA and CA or their ratio and different T lymphocyte functional subpopulations were sought. The results showed the presence of significant positive correlations between CA and CD4+/IFNγ+ T lymphocytes (R_Sp_ = 0.35, *p* = 0.02), as well as between the BA/CA ratio and CD4+/CD25^high^/Foxp3+ T lymphocytes (R_Sp_ = 0.35, *p* = 0.02) in MS patients. On the other hand, the BA/CA ratio was negatively correlated with CD4+/IFNγ+ T lymphocytes (R_Sp_ = −0.37, *p* = 0.01) in these same patients. Finally, BA was positively correlated with CD4+/CD25^high^/Foxp3+ T lymphocytes in HC (R_Sp_ = 0.53, *p* = 0.0006). These results are shown in Figure 3.

### Microbial Translocation and Gut Barrier Permeability

**Figure 4:**
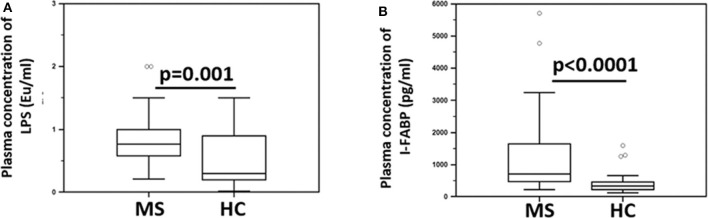
Indices of microbial translocation and indicators of alteration of the integrity of the gut barrier permeability in MS patients and healthy controls. Plasma concentrations of **(A)** LPS (Eu/ml) and **(B)** I-FABP (pg/ml) in Multiple Sclerosis patients (MS) (*n* = 38) and healthy controls (HC) (*n* = 38) were determined by ELISA. Comparisons between groups were performed using a two-tailed Mann-Whitney test for independent samples. The boxes stretch from the 25th to the 75th percentile; the line across the boxes indicates the median value; the lines stretching from the boxes indicate extreme values. Outliers are displayed as separate points. Statistical significance is shown.

Since BA is known to maintain the integrity of the gastrointestinal (GI) epithelial barrier, parameters of GI permeability were subsequently measured. The results showed that the plasma concentration of lipopolysaccharide (LPS) (median: HC = 0.3 Eu/ml; MS = 0.7 Eu/ml; *p* = 0.001) and intestinal fatty acid-binding protein (I-FABP) (median: HC = 336 pg/ml; MS = 715 pg/ml; *p* < 0.0001) were significantly increased in MS compared to HC (Figure 4). LPS translocates from the intestinal lumen to the peripheral circulation when the integrity of the GI barrier is altered; I-FABP is released into circulation in case of enterocyte damage and intestinal ischemia. These results therefore suggest the presence of damage to the gut barrier in MS patients.

### Gut Microbiota Layout in MS

**Figure 5:**
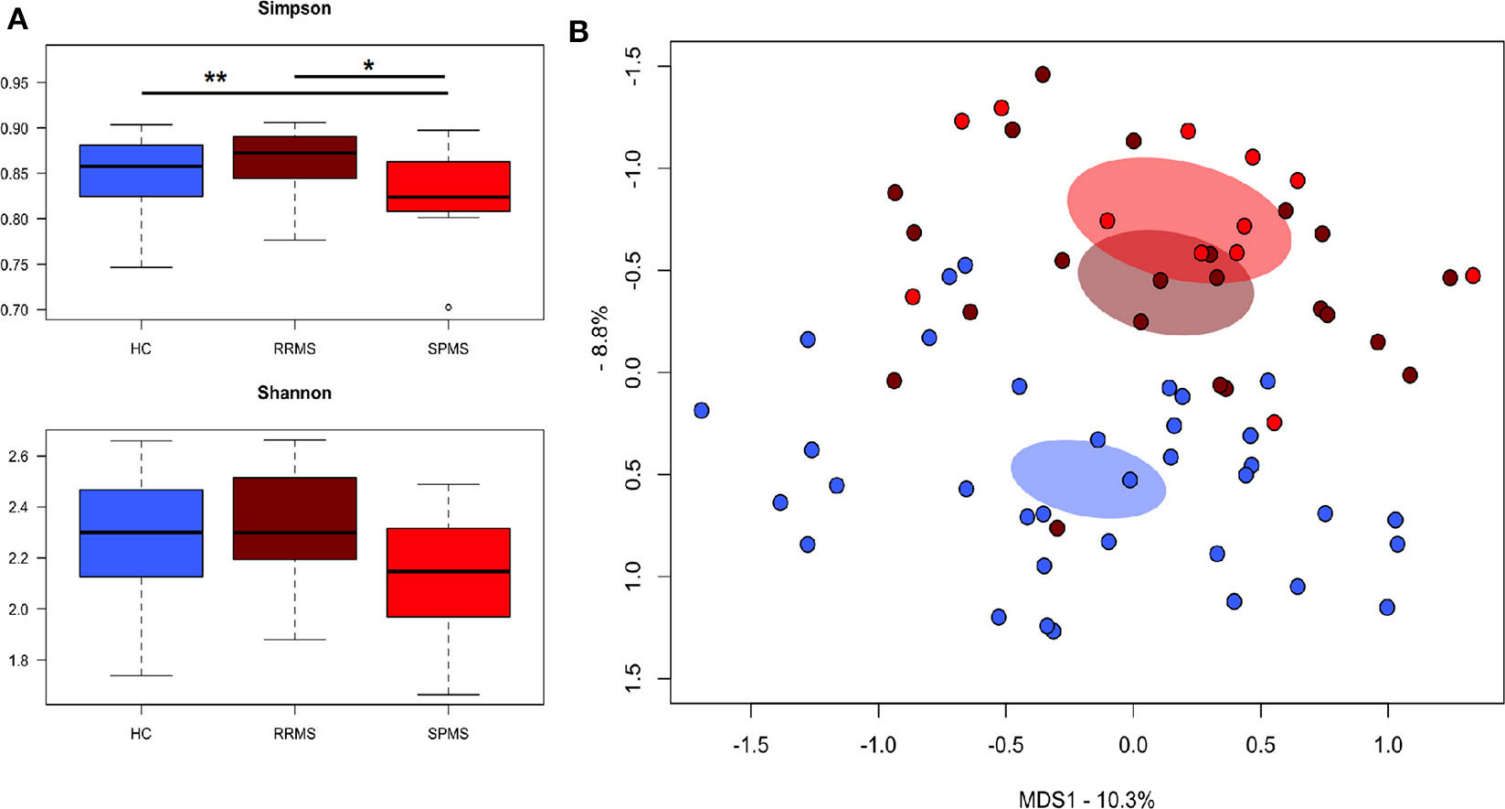
The gut microbiota of SPMS and RRMS patients segregates from that of healthy controls. **(A)** Boxplots showing the distribution of alpha diversity, measured using the Simpson (top panel) and Shannon (bottom panel) indices, for the gut microbiota of Multiple Sclerosis (MS) patients with secondary-progressive and relapsing-remitting disease (respectively, SPMS and RRMS). *p = 0.01; **p = 0.004; Wilcoxon test. **(B)** Principal Coordinates Analysis (PCoA) of the gut microbial communities, based on the Jaccard similarity index. A significant separation between MS patients and HC was found (*p* < 1 × 10^−4^, permutation test with pseudo-*F* ratios), but no significant differences were observed between SPMS and RRMS (*p* = 0.4).

**Figure 6:**
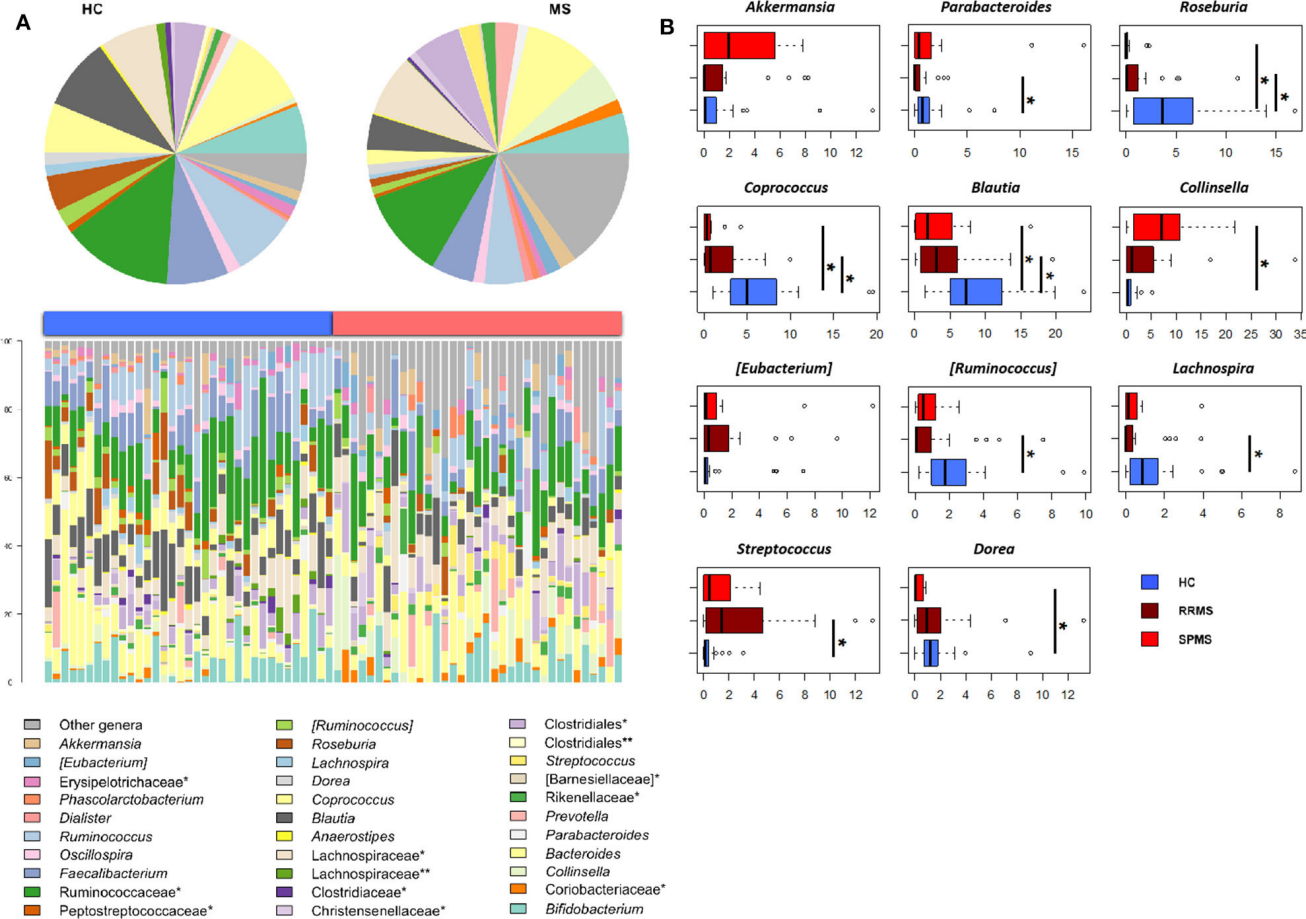
The dysbiotic layout of the gut microbiota in MS patients. **(A)** Genus-level relative abundance profiles of the gut microbiota of Multiple Sclerosis patients (MS, red) and healthy controls (HC, blue). Data are shown in the bar plots for each sample and in pie charts as average values. *, unclassified Amplicon Sequence Variants (ASVs) reported at higher taxonomic level; **, other unclassified ASVs. **(B)** Boxplots showing the relative abundance distribution of bacterial genera relevant for MS and significantly different between the study groups. *p ≤ 0.05; Wilcoxon test.

The gut microbiota (GM) of MS patients was finally profiled and compared with that of age- and sex-matched HC to verify whether the differences in SCFAs and MCFAs observed in these patients could be the result of changes in the gut microbial ecosystem. The 16S rRNA gene sequencing yielded a total of 1,520,873 high-quality reads, ranging from 23,759 to 80,331 per sample, clustered into 3,221 Amplicon Sequence Variants (ASVs). No differences in alpha diversity were observed between MS patients and HC, except for a slightly lower Shannon index value in the former (Supplementary Figure 1). On the other hand, when stratifying MS patients according to the severity of the disease, we found significantly lower diversity in secondary-progressive MS (SPMS) compared to relapsing-remitting MS (RRMS) and HC (inverse Simpson index; *p* ≤ 0.01, Wilcoxon test) (Figure 5A). Although not significant, a similar trend was observed for the Shannon index (*p* ≤ 0.1). The Principal Coordinates Analysis (PCoA) of inter-individual variation, based on the Jaccard similarity index, revealed a significant separation between MS patients and HC (*p* < 1 × 10^−4^, permutation test with pseudo-*F* ratios), while no significant differences were detected between MS subtypes (i.e., SPMS and RRMS) (*p* = 0.4) (Figure 5B and Supplementary Figure 1). In line with recent literature (7), the GM composition of MS patients showed increased relative abundance of *Akkermansia*, as well as a depletion of *Parabacteroides* (Figure 6A). Compared to HC, in the GM of MS patients we found a dramatic depletion of bacterial genera belonging to the *Lachnospiraceae* family. In particular, the proportions of the well-known SCFA producers *Roseburia, Coprococcus*, and *Blautia* were reduced by 4.5, 3.4, and 2 times, respectively, in the GM of MS vs. HC (*p* ≤ 0.02, Wilcoxon test) (Supplementary Figure 1). On the other hand, the MS microbiota showed a 7.4 and 2.4-fold increase in *Collinsella* and [*Eubacterium*], respectively (*p* ≤ 0.05) (Supplementary Figure 1). By focusing the analysis by disease subtype (Figure 6B), we found some commonalities, including the decrease in the SCFA producers mentioned above, but also specific microbial signatures. In particular, SPMS patients were found to be characterized by greater relative abundance of *Akkermansia* and *Collinsella* (*p* = 0.002), and a decrease in *Dorea* (*p* = 0.003). On the other hand, *Parabacteroides* was significantly under-represented in RRMS patients (*p* = 0.003), who also showed a reduction in [*Ruminococcus*] and *Lachnospira* (*p* ≤ 0.0008), a marked increase in *Streptococcus* (*p* = 0.006), and an increasing trend for [*Eubacterium*] (*p* = 0.07).

**Figure 7:**
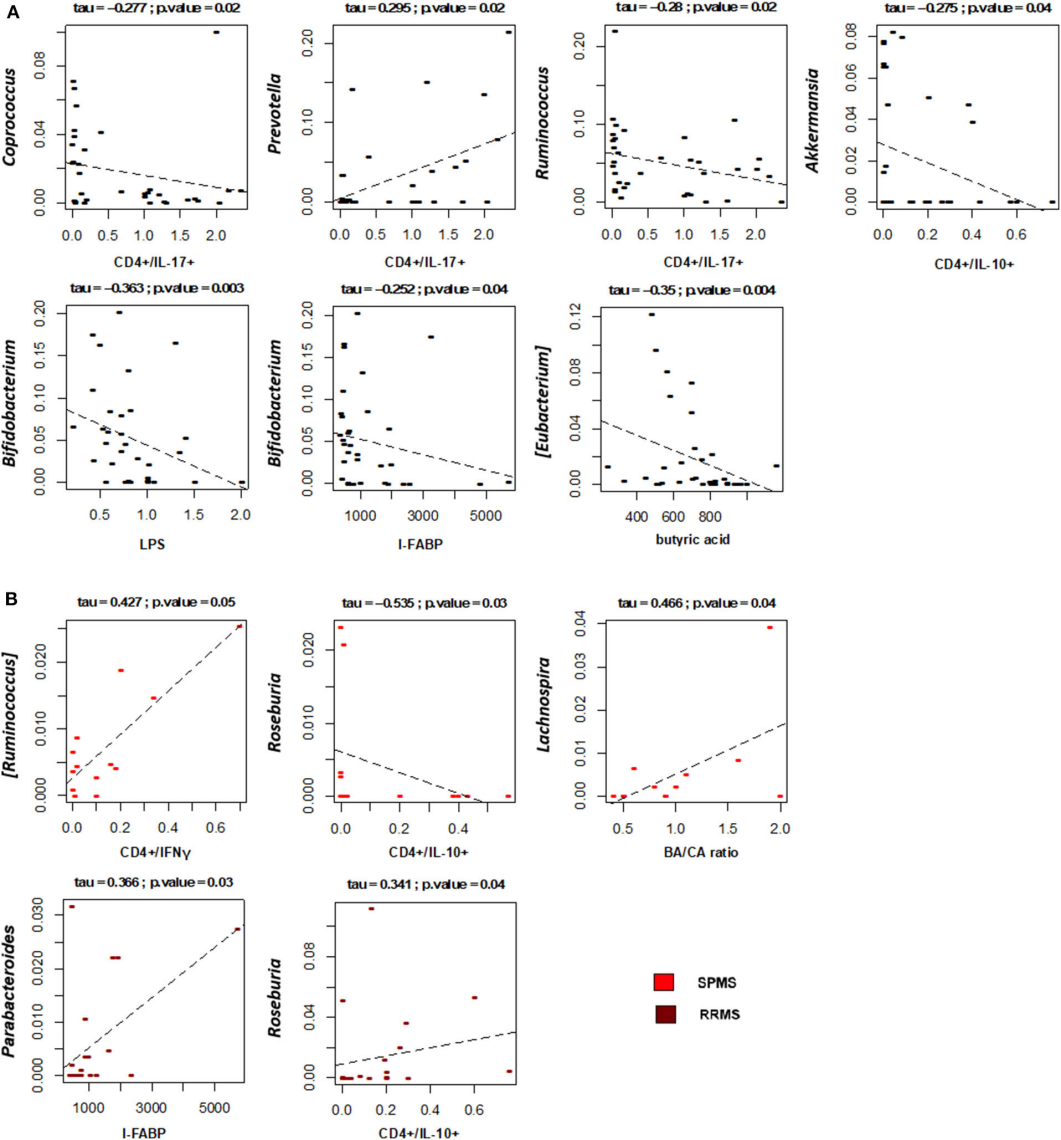
Associations between taxon relative abundances and levels of fatty acids, T cell subsets, LPS and I-FABP in MS patients. Only statistically significant correlations (*p* ≤ 0.05) based on Kendall rank correlation test are shown, for the entire cohort of Multiple Sclerosis (MS) patients **(A)** as well as for the two disease subtypes (SPMS, secondary-progressive MS and RRMS, relapsing-remitting MS) **(B)**.

Correlations between the relative abundances of bacterial taxa and EDSS, levels of T lymphocyte subpopulations, LPS and I-FABP in MS patients were next specifically sought (Figure 7A). Interestingly, the plasma levels of LPS and I-FABP were negatively correlated with the relative abundance of the well-known probiotic genus, *Bifidobacterium* (respectively, *p* = 0.003 and 0.04, tau = −0.363 and −0.252, Kendall rank correlation test). Inverse correlations were also found between the levels of CD4+/IL-17+ T lymphocytes and the proportions of the SCFA-producing genera, *Coprococcus* and *Ruminococcus* (*p* = 0.02, tau = −0.277 and −0.28, respectively). In contrast, a positive correlation was found between the levels of this T cell subpopulation and *Prevotella* (*p* = 0.02, tau = 0.295), whose relative abundance showed a 2.7-fold increase in MS compared to HC (2.9 ± 0.9 vs. 1.1 ± 0.6%), even if in the absence of statistical significance. Furthermore, levels of CD4+/IL10+ T lymphocytes were negatively correlated with *Akkermansia* (*p* = 0.04, tau = −0.275). As for fatty acids, a negative correlation was found between [*Eubacterium*] and the serum BA levels (*p* = 0.004, tau = −0.35). No significant correlations were found between the proportions of bacterial taxa and EDSS.

With specific regard to disease subtypes (Figure 7B), it is worth noting that in SPMS patients we found a positive correlation between CD4+/IFNγ+ T lymphocytes and [*Ruminococcus*] (*p* = 0.05, tau = 0.427), a well-known mucin-degrading gut microbe to be associated with Crohn's disease as well as other inflammatory disorders (51). A positive correlation was also found between the serum BA/CA ratio and *Lachnospira* (*p* = 0.04, tau = 0.466), consistent with the known ability of this bacterial genus to produce BA. As for RRMS, as expected based on available literature (52), *Roseburia* was found to positively correlate with the levels of CD4+/IL10+ T lymphocytes (*p* = 0.04, tau = 0.341). Another positive correlation was found between *Parabacteroides* and the serum levels of I-FABP (*p* = 0.03, tau = 0.366).

## Discussion

Multiple sclerosis (MS) is a chronic and progressive autoimmune disease characterized by inflammation whose etiopathogenesis is still unclear. Among the different hypothesis suggested to be involved in the pathogenesis of MS the inside-out model states that the disease is initially triggered by oligodendrocytes injury and/or death, possibly caused by oxidative stress (53). This would result in the release of myelin antigens into the periphery that would activate autoreactive B and T lymphocytes; these immune cells would migrate back into the central nervous system (CNS) and initiate inflammation (54). Inflammation is indeed observed throughout disease progression and is driven both by immune cell infiltration of the CNS and, directly, by CNS cells that are activated against the tissue damage that accumulates during the natural history of the disease.

Alterations in the composition of the gut microbiota have recently been held to be at least partially responsible for MS-associated inflammation (55). One additional set of results showed that butyrate-producing bacteria are reduced in MS (12). These bacteria are responsible for fermenting non-digestible carbohydrates in the proximal colon to generate SCFAs, including butyric acid (BA). The observation that BA drives Treg differentiation (56, 57) and maintains the integrity of the GI epithelial barrier (58–62), thus preventing microbial translocation and LPS-driven triggering of TLR4-mediated signaling, underlines the immunological importance of these bacteria and their byproducts, SCFAs, in down-modulating inflammation. BA can nevertheless induce inflammatory response as well (23, 63–65), and an elevated BA concentration in a proinflammatory milieu was shown to possibly result in stimulation of IFNγ-producing CD4+ T cells by inhibiting histone deacetylase activity (63), activating GPR41 or GPR43 (23, 64), or promoting survival of CD8+ T cells (65).

Even more recently, MCFAs, which are derived from the diet and can be generated by hepatic peroxisomal beta-oxidation of long-chain fatty acids, were shown to support the differentiation of TH1 and TH17 and to suppress that of Treg lymphocytes (29). Caproic acid (CA), in particular, seems to be prototypically endowed with such pro-inflammatory properties, which result from the activation of p38 MAPK signaling (29). Notably, CA can also be directly produced by some of the bacteria that form the microbiota. *Prevotella*, in particular, was shown to be able to generate CA. It is interesting to observe that these bacteria were indeed increased in MS patients, suggesting its possible role in the increased CA production seen in these patients.

Herein, we analyzed serum concentrations of BA and CA in a group of MS patients and observed that BA was significantly reduced and CA significantly augmented compared to healthy controls (HC). This peculiar SCFA/MCFA alteration correlated with the immunological profile expected to be supported by such a variation, i.e., an increase in TH1 and TH17 and a decrease in Treg lymphocytes, as it was observed in MS patients. The reduction in BA concentration had previously been described in MS patients with chronic progressive disease (66), and both effector and IL-10+ T cells were shown to be induced by SCFAs and dietary fiber in experimental autoimmune encephalomyelitis (EAE), the animal model of MS. These results suggested that modulation of SCFA production could represent a novel adjunct to therapeutic approaches in autoimmune CNS diseases (66).

Our data confirm and extend these observations by showing that the plasma concentration of both lipopolysaccharide (LPS) and intestinal fatty acid-binding protein (I-FABP) were significantly increased as well in MS patients compared to HC. LPS is a key component of the Gram-negative bacterial cell membrane; alterations of the intestinal mucosa result in the microbial translocation of such bacteria from the intestinal lumen into the systemic circulation with a consequent increase in LPS plasma concentration. I-FABP is a small (14–15 kDa) cytosolic protein found in mature enterocytes of the small and large intestine that is released when the integrity of the cell membrane is compromised (58). These results therefore suggest that the reduction of BA seen in MS is likely also responsible for impaired GI permeability (62). We do not know whether the augmented I-FABP concentrations observed in MS are the consequence of functional alterations of the small or of the large intestine; as SCFAs are produced in the large intestine it is nevertheless tempting to hypothesize that such I-FABP increments are the consequence of damages that affect the large intestine as a result of dysbiosis. Few studies address I-FABP in the setting of MS. Some data indicated that I-FABP as well as ileal bile acid binding protein (IBABP) are increased in MS patients, (67, 68), whereas other data did not observe any differences between patients and controls (69). Results herein lend support to the idea that intestinal damages, as indicated by alterations of LPS and I-FABP, are indeed present in MS; further analyses will be needed to sort out these discrepancies

The observed alteration of the SCFA/MCFA ratio could thus support MS-associated inflammation with at least two distinct mechanisms: a direct one, which is secondary to the skewing of T lymphocyte functional subset differentiation toward those subsets that support inflammation, and an indirect one driven by alterations in GI tract integrity and microbial translocation. As it has been extensively shown in the setting of another chronic and inflammatory disease, HIV infection (32), increased plasma levels of LPS lead to systemic immune activation and inflammation that is the consequence of LPS binding to TLR4, an activation protein expressed on different cell types. Notably, in EAE, the animal model of MS, alterations of the intestinal microbial ecosystem have also been shown to result in impaired permeability of the blood-brain barrier and activation of microglia and astrocytes (58).

Consistent with the alterations in the SCFA/MCFA ratio and supporting the above assumptions, the gut microbial ecosystem of MS patients was characterized by a dysbiotic layout that included: (1) a decrease in SCFA producers belonging to the *Lachnospiraceae* family, i.e., *Roseburia, Coprococcus*, and *Blautia*; (12) (2) an increased relative abundance of *Akkermansia*, a mucin degrader capable of inducing pro-inflammatory responses in human PBMCs and mono-colonized mice; (5, 7) (3) the depletion of *Parabacteroides*, a bacterial genus with anti-inflammatory properties shown to stimulate the maturation of IL-10–expressing CD4+CD25+ T cells in humans (7) and IL-10+Foxp3+ Tregs in mice; (7) and (4) an increased proportion of *Collinsella*, which has recently been shown to correlate with the production of the pro-inflammatory cytokine IL-17A and with altered gut permeability in MS (9). It is interesting to note that when we stratified patients based on the MS subtype (i.e., RRMS and SPMS), two distinct types of dysbiosis emerged. In particular, reduced microbial biodiversity and an overabundance of *Akkermansia* and *Collinsella* were specifically observed in patients with SPMS compared to HC. As briefly discussed above, these bacterial genera have already been associated with MS and shown to exacerbate its symptoms, because of their ability to induce pro-inflammatory responses and compromise the integrity of the intestinal epithelial barrier, thus contributing to aggravate a condition of chronic inflammation (5, 7, 9). On the other hand, the ecosystem configuration observed in patients with RRMS showed a different, somewhat less pronounced dysbiosis, with levels of diversity comparable to those of HC and mainly characterized by an overabundance of *Streptococcus* and depletion of *Parabacteroides*. It should be noted that this dysbiotic profile had already been found in cohorts of RRMS patients and suggested to influence the population of T lymphocytes and promote inflammation (4, 9, 70). In particular, the abundance of *Streptococcus* spp. has been shown to correlate positively with the proportion of TH17 cells while negatively with Tregs (71), thus representing a potential key factor in the development and/or reactivation of the disease.

CD4+/IL-17+ T lymphocytes were negatively correlated with *Coprococcus*, a bacteria known to be depleted in MS patients (72) and recognized as a beneficial commensal taxon, but correlated positively with *Prevotella*, whose role on the host physiology is instead more controversial. Some studies point in fact to its pro-inflammatory role in autoimmune diseases (73–75), while a very recent work indicates that *Prevotella histicola* can suppress EAE as efficiently as the disease-modifying drug Copaxone (76). This stresses the need for species-level analysis in future microbiome-based studies. On the other hand, the CD4+/IL-10+ T cell subpopulation was found to negatively correlate with *Akkermansia*, an organism that, as mentioned above, can exacerbate MS symptoms, probably either directly, by shifting immune responses toward a TH1 phenotype, or indirectly, by interacting with other bacteria and reducing their ability to drive Treg differentiation (77). In line with the available literature (52), the CD4+/IL-10+ T cell subpopulation was positively correlated with *Roseburia*, even if only in RRMS patients. On the other hand, in SPMS patients, CD4+/IFNγ+ T lymphocytes were found to positively correlate with [*Ruminococcus*], consistent with its ability to degrade mucus and induce inflammatory responses (51).

As for fatty acids, a negative correlation was found between serum BA levels and the relative abundance of [*Eubacterium*], a bacterial genus significantly increased in MS and comprising potential opportunistic pathogens that could affect the mucus layer (75, 78). On the contrary, a positive correlation was found between the serum BA/CA ratio and *Lachnospira* in SPMS patients, consistent with the known ability of this bacterial genus to produce BA. As expected, a negative correlation was also observed between the proportions of the health-promoting genus *Bifidobacterium* and serum levels of I-FABP and LPS. Several *in vitro* and *in vivo* studies in fact show that probiotics, including *Bifidobacterium* spp., are associated with improved barrier function and reduced metabolic endotoxemia (79, 80).

Overall, it is thus tempting to suggest that the disbyotic profile of the MS-associated gut microbiota justifies the SCFA/MCFA alterations described herein, possibly shedding light on the genesis of the inflammatory milieu that characterizes and accompanies MS. If this assumption is correct, then therapeutic/dietary interventions aimed at restoring a physiological (i.e., eubiotic) gut microbial ecosystem should be considered in MS therapy. The results obtained in the EAE animal model support this idea, since the administration of a multistrain probiotic has been shown to result in prophylactic and therapeutic efficacy that was associated with a reduced degree of inflammation (81, 82). Even more interestingly, in the same animal model, a SCFA-rich diet has been shown to increase the frequency of peripheral Tregs and ameliorate the clinical course of the disease, whereas mice given MCFA- or LCFA-rich diets showed an aggravated disease progression (29).

## Conclusions

Taken together, our results suggest a rationale explaining the etiology of MS-associated inflammation, possibly supporting the idea that alterations in the gut microbial ecosystem play a role in inflammatory autoimmune conditions, including MS, and reinforce the idea that interventions aimed at restoring microbiota eubiosis could be integrated into current therapeutic and rehabilitative strategies for MS. These are preliminary results and our study has limitations mostly stemming from the relatively small sample size and the heterogeneity of the enrolled patients in regard to treatment status. Additionally, we showed the presence of gut barrier damage and bacterial translocation in MS patients compared to controls, but the limited number of enrolled individuals did not allow us to verify possible differences in these parameters in patients with diverse disease phenotypes. Further studies will clarify these issues.

## Data Availability Statement

The datasets generated for this study are available on request to corresponding author. Sequence reads were deposited in the National Center for Biotechnology Information Sequence Read Archive (NCBI SRA; BioProject ID PRJNS633233).

## Ethics Statement

The studies involving human participants were reviewed and approved by The Ethics Committee of the Don Carlo Gnocchi Foundation. The patients/participants provided their written informed consent to participate in this study.

## Author Contributions

MC designed the research, drafted, and edited the manuscript. MS designed and performed research and drafted the manuscript. LM, LP, VR, and Ad'A selected patient groups, collected clinical data, organized patient enrolment, and collected blood sample. MB, ST, and PB performed gut microbiota experiments, analyzed the data, and drafted the manuscript. GR, EC, MDC, and RP performed chemical experiments, analysed, and wrote chemical data. FP, FL, and IM performed immunological experiments, analyzed results, and prepared the figures. All authors contributed to the article and approved the submitted version.

## Conflict of Interest

The authors declare that the research was conducted in the absence of any commercial or financial relationships that could be construed as a potential conflict of interest.

## Supplementary Material

Supplementary Figure 1 Gut microbiota configuration in MS patients. **(A)** Boxplots showing the distribution of alpha diversity, measured using the Simpson (top panel) and Shannon (bottom panel) indices, for the gut microbiota of Multiple Sclerosis patients (red) and healthy controls (blue). **(B)** Principal Coordinates Analysis (PCoA) of the gut microbial communities, based on the Jaccard similarity index. A significant separation between study groups was found (*p* < 1 × 10^−4^, permutation test with pseudo-*F* ratios). **(C)** Boxplots showing the relative abundance distribution of bacterial genera relevant for MS and significantly different between the study groups (*p* ≤ 0.05, Wilcoxon test).

Supplementary Table 1 Demographic and clinical data of the MS patients and the HC enrolled in the study.
