# Supplementary figures and images for: Alterations in Circulating Fatty Acid Are Associated With Gut Microbiota Dysbiosis and Inflammation in Multiple Sclerosis
Source: Front Immunol. 2020 Jul 7;11:1390. doi: 10.3389/fimmu.2020.01390 (PMC7358580; doi:10.3389/fimmu.2020.01390)

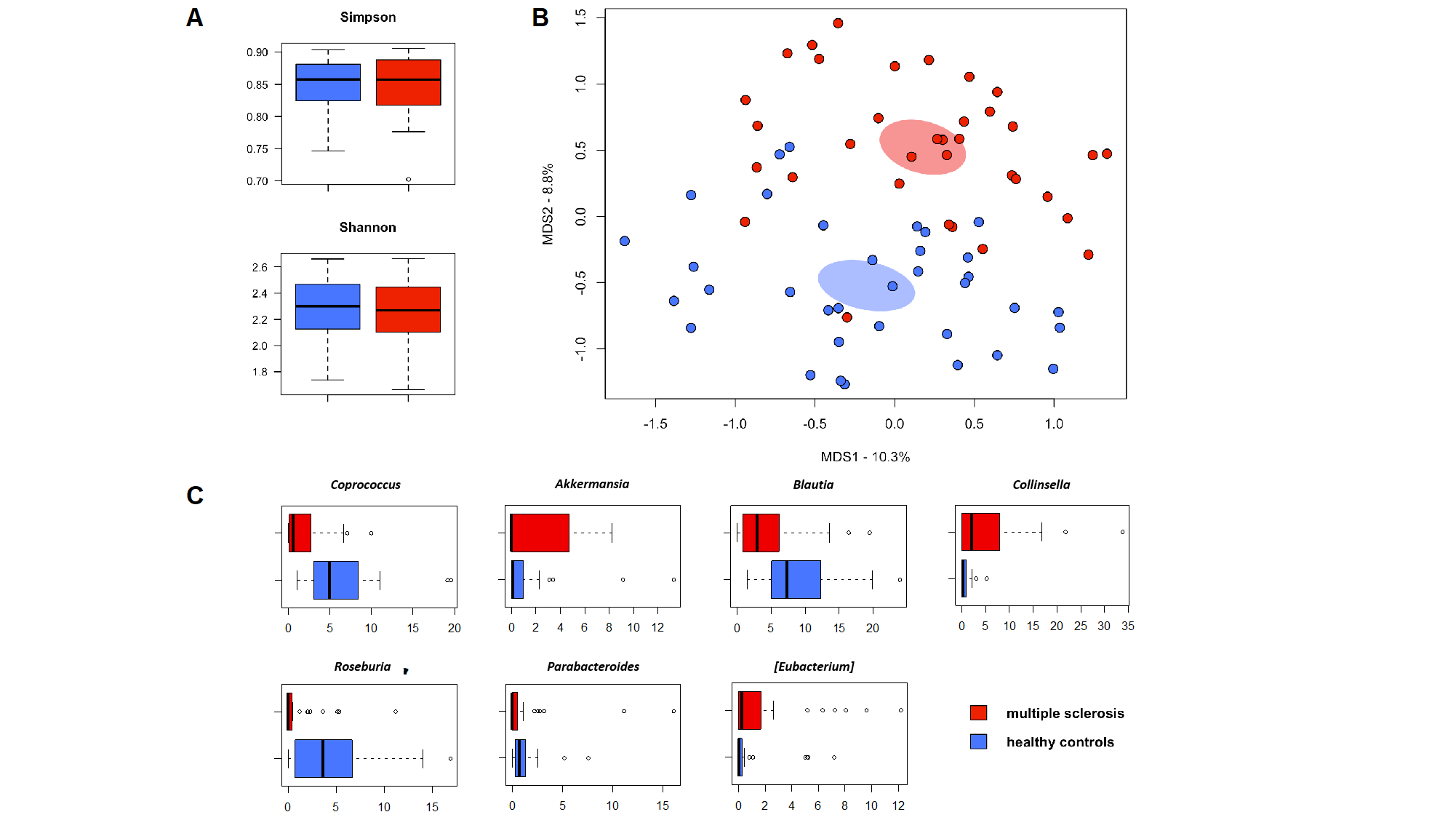

Supplement: Supplementary Figure 1 — Gut microbiota configuration in MS patients. (A) Boxplots showing the distribution of alpha diversity, measured using the Simpson (top panel) and Shannon (bottom panel) indices, for the gut microbiota of Multiple Sclerosis patients (red) and healthy controls (blue). (B) Principal Coordinates Analysis (PCoA) of the gut microbial communities, based on the Jaccard similarity index. A significant separation between study groups was found (p < 1 × 10^−4^, permutation test with pseudo-F ratios). (C) Boxplots showing the relative abundance distribution of bacterial genera relevant for MS and significantly different between the study groups (p ≤ 0.05, Wilcoxon test). [file Image_1.TIFF]
