# Supplementary material for: Alterations in Circulating Fatty Acid Are Associated With Gut Microbiota Dysbiosis and Inflammation in Multiple Sclerosis
Source: Front Immunol. 2020 Jul 7;11:1390. doi: 10.3389/fimmu.2020.01390 (PMC7358580; doi:10.3389/fimmu.2020.01390)
Supplement: Supplementary Table 1 — Demographic and clinical data of the MS patients and the HC enrolled in the study. [file Table_1.DOCX]

**Supplemental Table 1. Demographic and clinical data of the MS patients and the HC enrolled in the study**

|  | **MS total** | **MS RR** | **MS SP** | **HC** |
| --- | --- | --- | --- | --- |
| **N** | 38 | 26 | 12 | 38 |
|  |  |  |  |  |
| **Sex (M/F)** | 18/20 | 9/17 | 9/3 | 18/20 |
|  |  |  |  |  |
| **Age** (Median)  (IQ range) | 47  (42-57) | 44  (39-56) | 51  (45-60) | 48  (33-62) |
|  |  |  |  |  |
| **EDSS** (Median)  (IQ range) | 5.3  (3-6) | 4  (1.9-5.3) | 6.5  (6.0-6.6) | N/A |
|  |  |  |  |  |
| **Disease Duration** (Median)  (IQ range) | 19  (15-24) | 17.8  (14.8-21.3) | 21.9  (11.9-32.4) | N/A |
|  |  |  |  |  |
| **DMT** |  |  |  | N/A |
| **Copaxone** | 7 | 6 | 1 | N/A |
| **Aubagio** | 2 | 2 | 0 | N/A |
| **Betaferon** | 2 | 1 | 1 | N/A |
| **Tecfidera** | 1 | 1 | 0 | N/A |
| **Fingolimod** | 1 | 1 | 0 | N/A |
| **Natalizumab** | 1 | 1 | 0 | N/A |
| **Lemtrada** | 1 | 0 | 1 | N/A |
| **Rituximab** | 1 | 1 | 0 | N/A |
|  |  |  |  |  |
| **No DMT** | 22 | 13 | 9 | N/A |
